# Agmatine improves liver function, balance performance, and neuronal damage in a hepatic encephalopathy induced by bile duct ligation

**DOI:** 10.1002/brb3.3124

**Published:** 2023-06-20

**Authors:** Sepideh Ganjalikhan‐hakemi, Majid Asadi‐Shekaari, Fahimeh Pourjafari, Gholamreza Asadikaram, Masoumeh Nozari

**Affiliations:** ^1^ Student Research Committee, Department of Anatomical Sciences, Afzalipour School of Medicine Kerman University of Medical Sciences Kerman Iran; ^2^ Neuroscience Research Center, Institute of Neuropharmacology Kerman University of Medical Sciences Kerman Iran; ^3^ Department of Biochemistry, Afzalipour School of Medicine Kerman University of Medical Sciences Kerman Iran

**Keywords:** agmatine, behavior, bile duct ligation (BDL), hepatic encephalopathy

## Abstract

**Introduction:**

In the current study, we investigate whether oral administration of agmatine (AGM) could effectively reduce motor and cognitive deficits induced by bile duct ligation (BDL) in an animal model of hepatic encephalopathy (HE) through neuroprotective mechanisms.

**Methods:**

The Wistar rats were divided into four groups: sham, BDL, BDL+ 40 mg/kg AGM, and BDL+ 80 mg/kg AGM. The BDL rats were treated with AGM from 2 weeks after the surgery for 4 consecutive weeks. The open field, rotarod, and wire grip tests were used to assess motor function and muscle strength. The novel object recognition test (NOR) was performed to evaluate learning and memory. Finally, blood samples were collected for the analysis of the liver markers, the animals were sacrificed, and brain tissues were removed; the CA1 regions of the hippocampus and cerebellum were processed to identify apoptosis and neuronal damage rate using caspase‐3 immunocytochemistry and Nissl staining.

**Results:**

The serological assay results showed that BDL severely impaired the function of the liver. Based on histochemical findings, BDL increased the neuronal damage in CA1 and Purkinje cells, whereas apoptosis was significantly observed only in the cerebellum. AGM treatment prevented the increase of serum liver enzymes, balance deficits, and neuronal damage in the brain areas. Apoptosis partially decreased by AGM, and there were no differences in the performance of animals in different groups in the NOR.

**Conclusions:**

The study suggests AGM as a potential treatment candidate for HE because of its neuroprotective properties and/or its direct effects on liver function.

## Graphical abstract

Agmatine prevented the increase of serum liver enzymes induced by chronic cholestasis. Agmatine treatment improved balance deficits, and neuronal damage induced by chronic cholestasis.

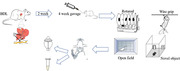

## 1 INTRODUCTION

Hepatic encephalopathy (HE) is a cerebral dysfunction syndrome secondary to acute or chronic liver diseases. The syndrome is characterized by a wide spectrum of neuropsychiatric manifestations ranging from mild subclinical alterations to coma (Dhanda et al., 2013). Various grades of mental and motor disturbances, including personality changes, poor concentration, attention, and judgment, learning and memory deficits, anxiety, fatigue, slow movements, confusion, and lethargy, were associated with HE. These changes affect the quality of life in patients and decrease their ability to perform work and daily life activities (Méndez et al., 2011; Monfort et al., 2009; Munoz, 2008).

Despite considerable experimental efforts, the pathophysiology of HE is still largely unknown. Hyperammonemia is the main cause of HE, which disrupts the blood–brain barrier and exerts a prominent toxic effect on brain tissue (Braissant et al., 2013; Jiang et al., 2013). In the liver, ammonia is converted to urea through the urea cycle, which is water soluble and can be excreted by the kidneys. The first step of the urea cycle is converting NH4^+^ to carbamoyl phosphate by mitochondrial carbamoyl phosphate synthetase I (CPS‐1). *N*‐acetyl glutamate (NAG) is an essential allosteric cofactor of CPS‐1, and its availability increases ureagenesis. Previous studies showed that high‐protein diets and arginine could increase NAG synthesis and promote the urea cycle. It was even reported that agmatine (AGM), a metabolite of arginine, can increase NAG more than arginine. AGM was introduced as an effective therapeutic adjunct in some urea cycle disorders and toxic hyperammonemia (Dhanda & Sandhir, 2018; Nissim et al., 2002; Savlan et al., 2014).

Oxidative stress and inflammation are other causes of developing liver disease and HE; antioxidants and anti‐inflammatories have been extensively suggested as treatment strategies. The anti‐inflammatory, antioxidative, and neuroprotective effects of AGM have been approved frequently. A recent study has reported the therapeutic effects of AGM on hepatic and renal injury in an experimental model of obstructive jaundice and has related these positive effects to the antioxidant properties of AGM (Ommati et al., 2020).

NMDA‐receptor (*N*‐methyl‐d‐aspartate) activation contributes to the pathogenesis of HE and has been shown that NMDA‐receptor antagonists are of potential therapeutic value in the treatment of this syndrome. AGM is an endogenous NMDA antagonist, and a neurotransmitter/neuromodulator with some cognitive protective effects, such as anxiolytic, antidepressant, and memory‐enhancing activity (Aglawe et al., 2021; Ahmadi et al., 2015; Kim et al., 2016; Nissim et al., 2002; Su et al., 2009; Taksande et al., 2014).

Based on the mentioned hypotheses in the etiology of HE and the positive functions of AGM in this approach, the present study was designed to explore the effects of AGM in bile duct ligated (BDL) rats, an animal model of HE. For these purposes, the motor and cognitive behaviors of the experimental animals were assessed along with providing histopathological evidence.

## 2 MATERIALS AND METHODS

### 2.1 Animal

In this study, 45 male adult Wistar rats were used in the weight range of about 200–250 g and kept in a 12/12 dark/light cycle (lights on at 7:00 a.m.). All procedures were done on the animals that were approved by the Kerman University of Medical Sciences Ethics Committee (IR.KMU.AH.REC.1400.018). During the study period, animals had free access to food and water.

### 2.2 Bile duct ligation (BDL) surgery

Animals were anesthetized by intraperitoneal administration of ketamine (100 mg/kg) and xylazine (20 mg/kg). The abdominal wall was incised in the middle line, and common bile duct was seen and ligated with a nonabsorbable suture by two ligatures. The first ligature was tied below the junction of the hepatic ducts, and the second was above the entrance of the pancreatic ducts. The common bile duct was cut completely between the ligatures (BDL rats). In sham operation group, animals underwent operation without BDL ligation and cutting (Ahmadi et al., 2015). The percentage of mortality before the start of treatment (2 weeks after BDL surgery) was about 50%.

### 2.3 Drug treatments

**FIGURE 1:**
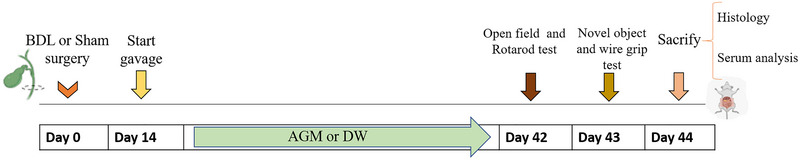
The schedule of this study. BDL (bile duct ligation), DW (Distilled water).

Two weeks after surgery, the BDL animals were randomized into three groups that were gavaged with two different doses of AGM (40 and 80 mg/kg) or distilled water (DW) daily, for 4 weeks. AGM was purchased from Sigma (A7127 Sigma‐Aldrich) and dissolved in DW. The sham animals were gavaged with the same volume of DW. The final study groups were (1) sham; (2) BDL; (3) BDL+ AGM 40 mg/kg; (4) BDL+ AGM 80 mg/kg. Six weeks after surgery (after 4 weeks of the drug treatment), sham and BDL rats underwent open field, rotarod, wire grip, and novel object recognition (NOR) test to evaluate the locomotor activity, balance, muscle strength, and cognitive impairment (Li et al., 2003; Ommati et al., 2020; Sharawy et al., 2018). The schedules of the experiments are summarized in Figure 1.

### 2.4 Behavioral studies

#### 2.4.1 Open‐field test

Animals motor activity was assessed by an open‐field test. The apparatus consisted of a square plexiglass box with dimensions of 90 × 90 × 30 cm^3^. The floor of arena is divided into 16 small squares by imaginary lines. At the beginning of the procedure, each rat was placed in the center of the box and allowed to move freely for 5 min; its activity was recorded automatically. The following behavioral parameters were scored by a video tracking system (BORJ SANAT, Iran, http://borjsanat.ir). An experimenter blind to animal status also scored the number of vertical locomotor activities (rearing and climbing) and grooming (rubbing the body and head). After each animal, the chamber was cleaned with a solution of 70% ethanol (Mohammadi et al., 2020).

#### 2.4.2 Rotarod test

We used an accelerating rotating rod (Tajhiz Gostar‐e Omid Iranian Co.) to evaluate motor function and coordination. The protocol was as Ghotbi Ravandi et al. (2019) reported previously. The animals were placed on a rotating horizontal rod with a 3 cm diameter and rotated at a minimum speed of 10 rpm (revolutions per minute) to a maximum speed of 60 rpm. The test session consisted of three trials (intertrial interval = 15 min, cut‐off: 300 s), during which the time on the road that animals were able to maintain their balance was recorded (Ghotbi Ravandi et al., 2019; Shabani et al., 2012).

#### 2.4.3 Wire grip test

A wire grip test was performed to evaluate neuromuscular strength. Each rat was hung vertically with its front legs on a horizontal steel wire with a length of 80 cm and a diameter of 7 mm, and latency to fall was recorded. The test was done for three trials for each animal, and the average time was reported; the intertrial rest interval was 10 min (Haghani et al., 2013).

#### 2.4.4 Novel object recognition test

A NOR test, which assesses the non‐rewarded, nonspatial memory, was chosen to evaluate cognitive impairment induced by BDL and the possible protective effects of AGM. The test was performed in a plexiglass box (40 × 60 × 60 cm^3^). A camera connected to a computer equipped with a video tracking system recorded the animal's behavior. The task was performed during three stages: habituation, training, and retention. The day before the test, each animal was placed in the empty NOR box without objects for habituation and allowed to freely explore it for 5 min. In the training stage, two objects (A: green and B: black) with the same volume and size were placed in the corners of the NOR box at a certain distance from the wall, and the rats were allowed to explore items for 5 min. After 45 min, in the retention stage, one of the objects (B: black) was replaced by a new object (C: red). The animal's exploratory behaviors, including pointing the nose to an object at a distance of 2 cm or touching it with the nose or forehead, were recorded for 3 min. The objects and box were cleaned with alcohol between the experiments. A preference index (in the retention stage) was calculated as the ratio between the time spent exploring the novel object (object C) to the total time spent in exploring both the objects (objects A and C) and similar for object B in training phase was calculated (Faatehi et al., 2019).

### 2.5 Liver function tests

After the behavioral test, the animals were anesthetized, and blood was drawn from the apex of the heart. Blood samples were centrifuged, and plasma was separated and stored frozen at −20°C until assayed. Liver enzymes, including alanine aminotransferase (ALT), aspartate aminotransferase (AST), alkaline phosphatase (ALP), and total and direct bilirubin, are measured to determine the hepatic injury. We also calculated AST to ALT ratio (AST/ALT) because AST and ALT are not in parallel during liver damage and clinical studies suggest AST/ALT as a useful predictor for liver injury. Standard kits (Pars Azmun, Tehran, Iran) and an autoanalyzer Selectra XL (Vital Science, the Netherlands) were used to determine liver function markers (Botros & Sikaris, 2013).

### 2.6 Histological studies

Immediately after collecting the blood samples, transcranial perfusion was conducted, first with isotonic saline followed by 4% paraformaldehyde (PFA) and 0.1 M phosphate buffer saline (PBS). Then brains were dissected and postfixed with 4% PFA/0.1 M PBS overnight. After paraffin embedding, 5‐μm sagittal sections were prepared for further investigation of histological staining and immunohistochemistry studies.

#### 2.6.1 Nissl staining

In this method, briefly, sections were deparaffinized with xylene, rehydrated with gradient alcohol, and then stained with 0.1% thionin (Sigma, St. Louis, MO, USA) solution for 5–10 min and mounted with Entellan. CA1 regions of the hippocampus and cerebellum were observed for the assessment of neurodegeneration induced by BDL. Neurons with a clear round nucleus and intact cytoplasm membrane were counted as intact neurons. However, neurons with shrunken cell bodies, pyknotic nuclei, and dark cytoplasm were considered damaged neurons (Mohammadi et al., 2020).

#### 2.6.2 Caspase‐3 immunohistochemical staining

After deparaffinization and rehydration, slides were washed three 5‐min with phosphate buffer saline (PBS) and boiled in sodium citrate buffer (pH 6.0) for 20 min. They were cooled to room temperature and incubated in 3% hydrogen peroxide for 30 min and washed again. After that, the sections were blocked with 10% goat serum and 0.4% Triton‐X100 and incubated with the caspase‐3 primary antibody (1:50; sc‐5652 Santa Cruz) at 4°C overnight. After rinsing (three times in PBS), the slides were incubated with an anti‐rabbit secondary antibody conjugated with horse‐radish peroxidase for 1 h at room temperature. The immunoreactivity was visualized with DAB (3,3′‐diaminobenzidine) for 15 min. Finally, the nuclei were stained with hematoxylin and mounted. Images were taken using a microscopic digital camera system. The number of caspase‐3‐positive neurons in the cerebellum and CA1 region of the hippocampus (four animals/group, two slides/rat, and four fields/slide) was counted by an observer blinded to the experiment (Pourjafari et al., 2021).

### 2.7 Statistical analysis

Data were analyzed using GraphPad Prism software (version 8.4.3, GraphPad Software, San Diego, California USA). The normality and homogeneity of variances were investigated with the Shapiro–Wilk test and the Levene test, respectively. Analysis was then performed by one‐way ANOVA followed by Tukey's multiple comparisons post hoc (for data that passed normality tests) or by nonparametric Kruskal–Wallis test followed by Dunn's posttest (for data that did not pass normality tests). We also used a repeated‐measure ANOVA to assess the BDL and AGM effects on motor learning during three trials. *p* < .05 was considered significant. Data are expressed as min to max or mean ± SEM.

## 3 RESULT

### 3.1 Assessment of jaundice following BDL

**FIGURE 2:**
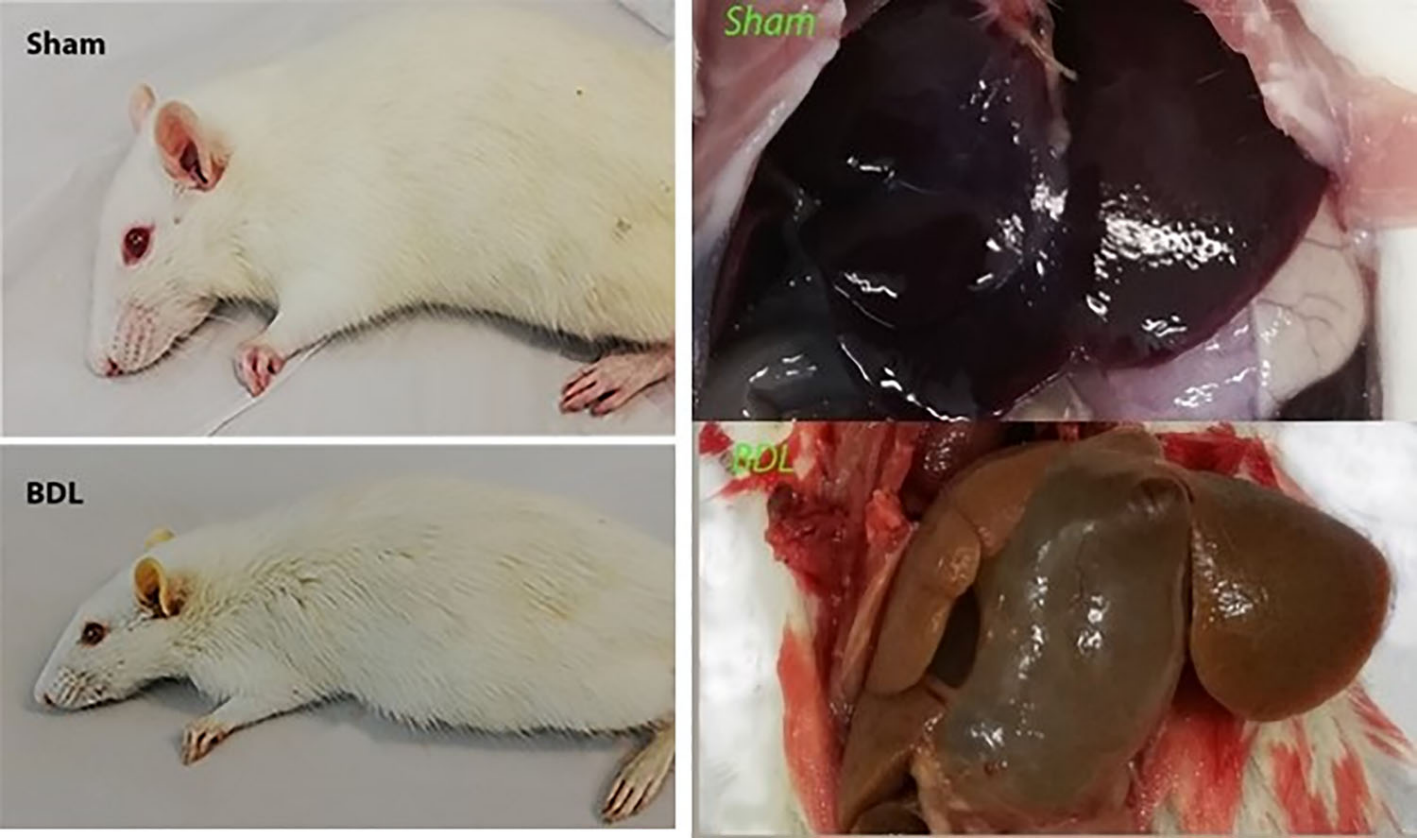
Yellow ears, skin, paws, tail, and liver, plus enlarged abdomen in bile duct ligation (BDL) rats compared to sham rats.

The general appearances of rats (after 6 weeks of the surgery) and their livers (the end of the study) were assessed and shown in Figure 2. The BDL rats had yellow liver, ears, sclera, skin, paws, and tails, as well as an enlarged abdomen.

### 3.2 Assessment of liver function tests

**FIGURE 3:**
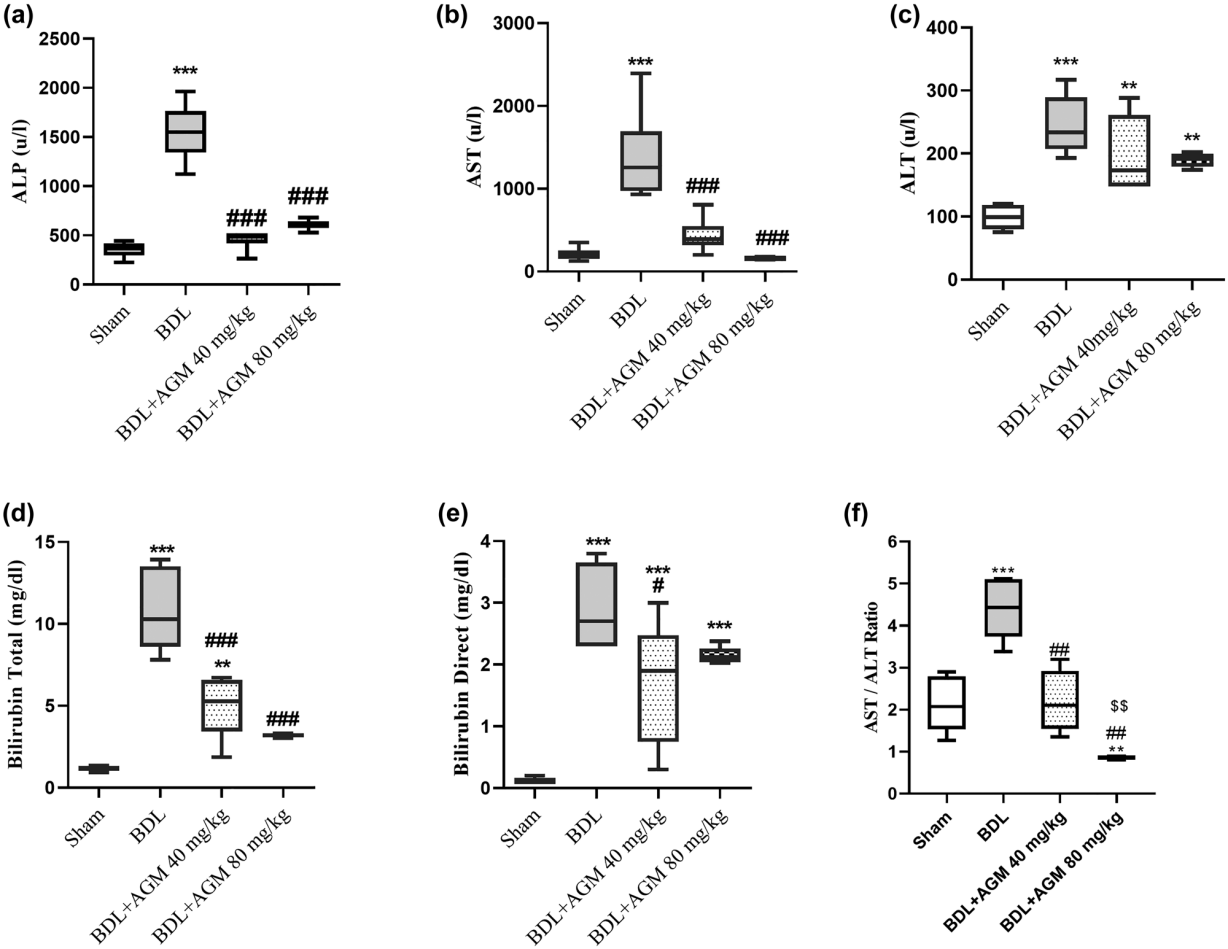
Analysis of serum biochemical parameters following bile duct ligation (BDL) and agmatine (AGM) (40 and 80 mg/kg) treatment in male rats. Alkaline phosphatase (ALP) (a); Aspartate aminotransferase (AST) (b); Alanine aminotransferase (ALT) (c); Bilirubin total and direct (d and e); AST to ALT ratio (AST/ALT). ^**^ *p* < .01, and ^***^ *p* < .001 versus sham; ^#^ *p* < .05, ^###^ *p* < .001 versus BDL, ^$$^ *p* < .01 versus BDL+ AGM 40 mg/kg. Data are shown as box plots (Whiskers: min to max), (*n* = 6).

As demonstrated in Figure 3, the plasma level of liver enzymes and bilirubin (total and direct) showed a significant increase in the BDL group compared with the sham group (ALP: *p* < .001, Figure 3a, AST: *p* < .001, Figure 3b; ALT: *p* < .001, Figure 3c; total and direct bilirubin: *p* < .001, Figure 3d,e).

Treating with 40 mg/kg AGM significantly decreased ALP (*p* < .001, Figure 3a), AST (*p* < .001, Figure 3b), bilirubin total (*p* < .001, Figure 3d), and direct bilirubin (*p* = .012, Figure 3e) compared with BDL rats.

AGM at doses of 80 mg/kg significantly reduced ALP (*p* < .001, Figure 3a), AST (*p* < .001, Figure 3b), and bilirubin total (*p* < .001, Figure 3d) compared to the BDL group.

The results of comparisons between multiple experimental groups were as follows: ALP: (*F* (3,20) = 71.79, *p* < .001), ALT: (*F* (3,20) = 14.28, *p* < .001), AST: (*F* (3,20) = 22.66, *p* < .001), bilirubin total (*F* (3,20) = 42.89, *p* < .001), bilirubin direct (*F* (3,20) = 23.61, *p* < .001).

One‐way ANOVA analysis showed statistically significant difference between the groups in the ratio of AST/ALT (*F* (3,20) = 36.34, *p* < .001), and Tukey's post hoc test confirmed a significant increase in this ratio following BDL‐induced HE (*p* < .001, Figure 3f). Both doses of AGM (40 and 80 mg/kg) significantly reduced the ratio compared with the BDL rats (*p* < .001, Figure 3f). Elevated AST/ALT ratio is associated with increased risk of developing fibrosis.

### 3.3 Assessment of locomotor activity

Impairments of locomotor activity and muscle strength are associated with several liver diseases and have been reported in BDL model of cirrhotic and HE. In this study, we evaluated these parameters by using open field, rotarod, and wire grip test and obtained the following results.

#### 3.3.1 Open field

**FIGURE 4:**
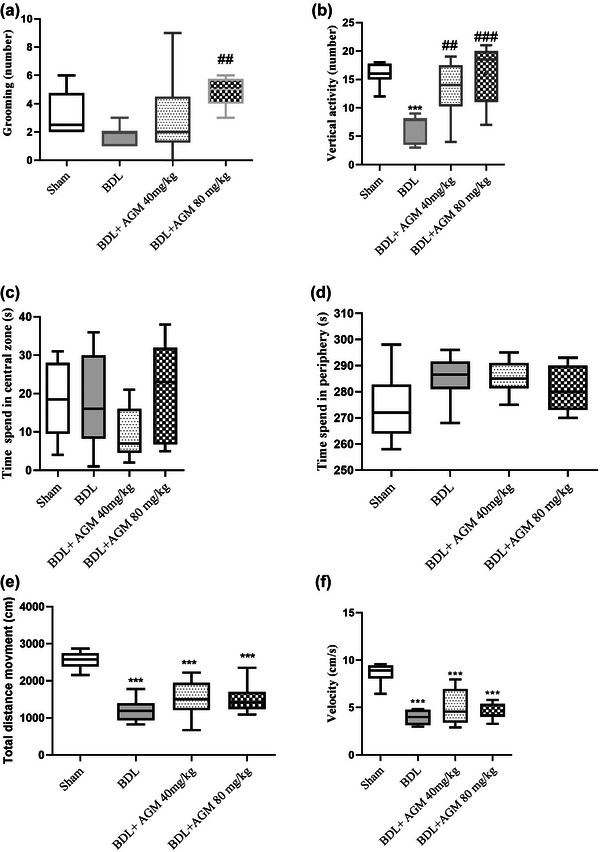
Locomotor activity following bile duct ligation (BDL) and agmatine treatment (40 and 80 mg/kg) in male rats. Grooming and vertical activity (a and b); time spent in the central (c) and peripheral (d) areas; total distance moved (e), and velocity (f). ^***^ *p* < .001. Compared with sham group, ^##^ *p* < .01, ^###^ *p* < .001 compared with BDL. Data are shown as box plots (Whiskers: min to max), (*n* = 8).

In the open‐field test, grooming behavior in BDL rats was not change (*F* (3,28) = 4.01, *p* = .32, Figure 4a), whereas vertical activity, including climbing and rearing in BDL rats, was less than sham‐operated rats (*F* (3,28) = 10.59, *p* = .0003. Figure 4b). AGM at both doses increased this type of exploratory behavior (BDL+ AGM 40 mg/kg: *p* = .008, BDL+ AGM 80 mg/kg: *p* = .0002 compared with BDL group). We did not observed significant difference between the groups in time spent in the central (*F* (3,28) = 1.70, *p* = .18, Figure 4c) and peripheral (*F* (3,28) = 2.54, *p* = .07, Figure 4d) areas of the open field. The total distance moved (*F* (3,28) = 19.81, *p* < .001, Figure 4e) and velocity (*F* (3,28) = 24.53, *p* < .001, Figure 4f) were decreased in all groups, compared to sham group.

#### 3.3.2 Rotarod

**FIGURE 5:**
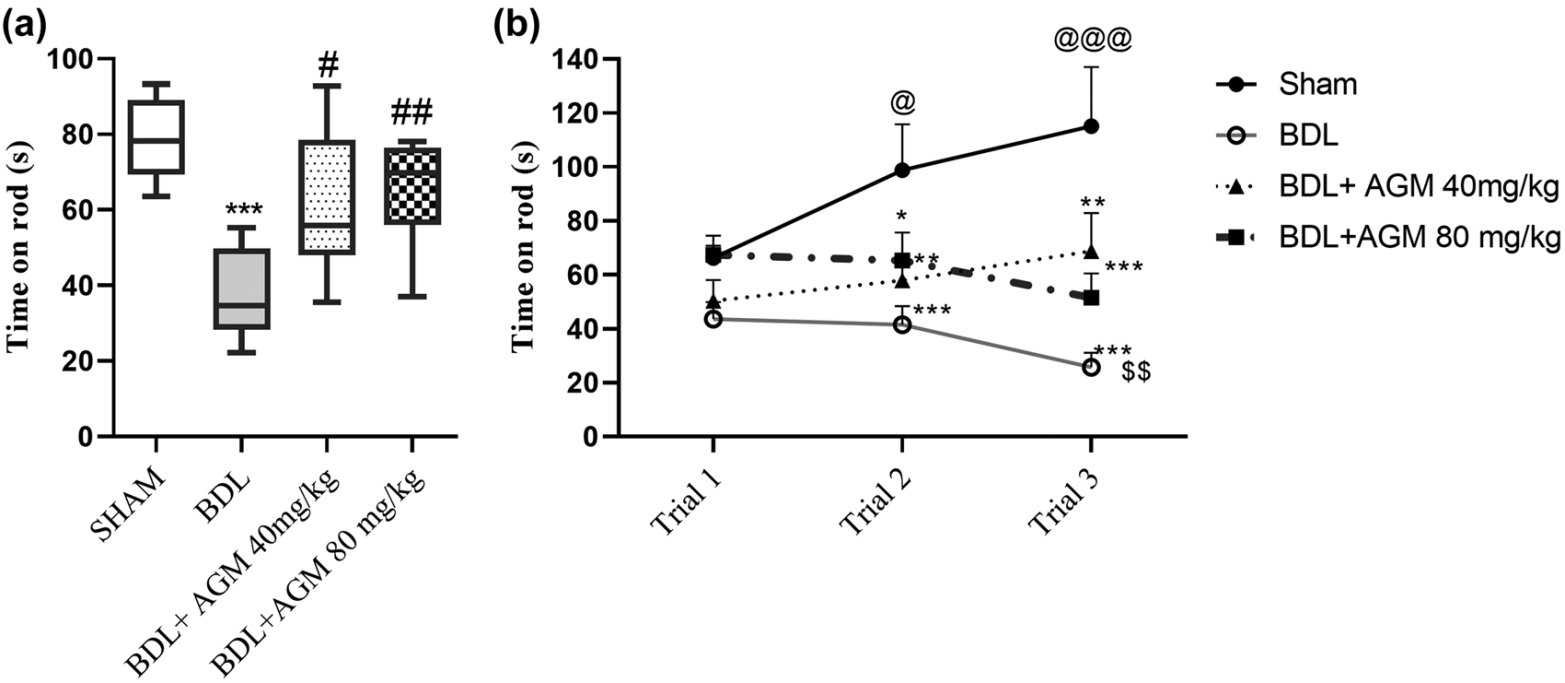
Motor coordination and learning following bile duct ligation (BDL) and agmatine (AGM) treatment (40 and 80 mg/kg) in male rats. Mean time on rotarod of three trials (a), rotarod skill acquisition in three successive trials for all groups (b). ^*^ *p* < 0.05, ^**^ *p* < 0.01, and ^***^ *p* < 0.001 versus sham; ^#^ *p* < 0.05, ^##^ *p* < 0.01 versus BDL; ^$$^ *p* < 0.01 compared with BDL+ AGM 40 mg/kg; ^@^ *p* < 0.05, @@@*p* < 0.001 versus the first trial within each group (*n* = 7).

One‐way ANOVA analysis showed a statistically significant difference between the groups in the mean latency of three trials on accelerating rotarod (time on rod: *F* (3,28) = 11.75, *p* < .001, Figure 5a), and Tukey's post hoc test showed a significant decrease in this parameter following BDL‐induced HE compared with sham animals (*p* < .001, Figure 5a). BDL rats stayed on the rotarod longer when they received 40 or 80 mg/kg AGM (BDL+ AGM 40: *p* = .01; BDL+ AGM 80: *p* = .002 vs. BDL).

Repeated measure ANOVA showed that only sham animals were able to learn the rotarod task and indicated a significant improvement in task acquisition (the first trial compared to the second [*p* < .05] and third trial [0.001]; *F* (3,21) = 7.8, *p* = .001 for sham groups, Figure 5b). There was a significant difference in the performance of different groups with sham group in the second (BDL: *p* < .001; BDL+ AGM 80: *p* < .01; BDL+ AGM 40: *p* < .05) and third trial (BDL: *p* < .001; BDL+ AGM 80: *p* < .001; BDL+ AGM 40: *p* < .01). BDL+ 40 mg/kg AGM rats stay on the road longer than BDL rats in the third trial (*p* < .01).

#### 3.3.3 Wire grip

**FIGURE 6:**
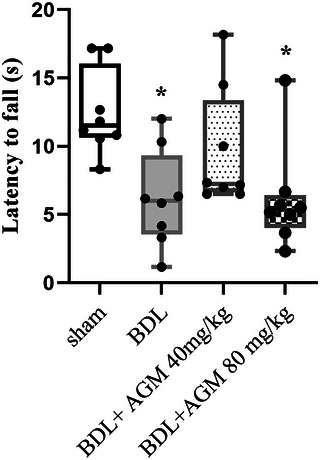
Mean latency to fall using wire grip test following bile duct ligation (BDL) and agmatine treatment (40 and 80 mg/kg) in male rats. ^**^ *p* < 0.01 versus sham, data are shown as box plots (Whiskers: min to max), (*n* = 8).

The muscle strength and balance in the wire grip test were significant among different groups (Kruskal–Wallis statistic = 14.19, *p* = .002). Our results demonstrate a significant decrease in latency to fall in BDL rats (*p* = .01, BDL vs. sham, Figure 6). There was no significant difference among the BDL+ 40 mg/kg AGM animals and sham rats (*p* > .05).

### 3.4 Assessment of learning and memory

**FIGURE 7:**
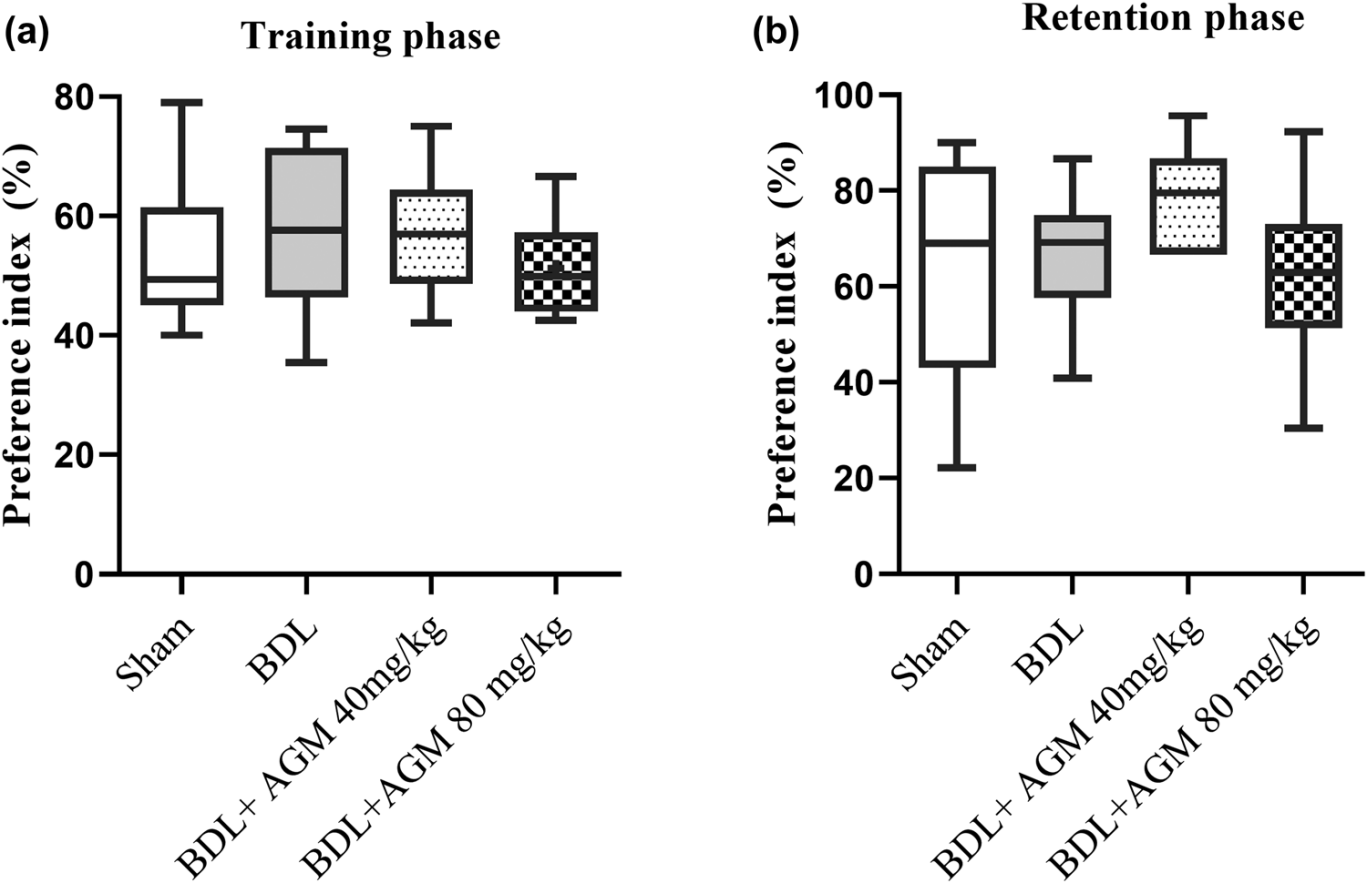
Learning and memory assessment using novel object recognition test following bile duct ligation (BDL) and agmatine treatment (40 and 80 mg/kg) in male rats. Preference index in training (a) and retention sessions (b).

Learning and memory were assessed using the NOR test. No significant change was observed in both training (*F* (3,20) = 0.38, *p* = .76, Figure 7a) and retention phase (*F* (3,20) = 0.96, *p* = .42, Figure 7b). Animals had no preference for either object in training sessions. Three animals in the BDL group did not move at all and were removed from the analysis.

### 3.5 Assessment of histological features

#### 3.5.1 Nissl staining

**FIGURE 8:**
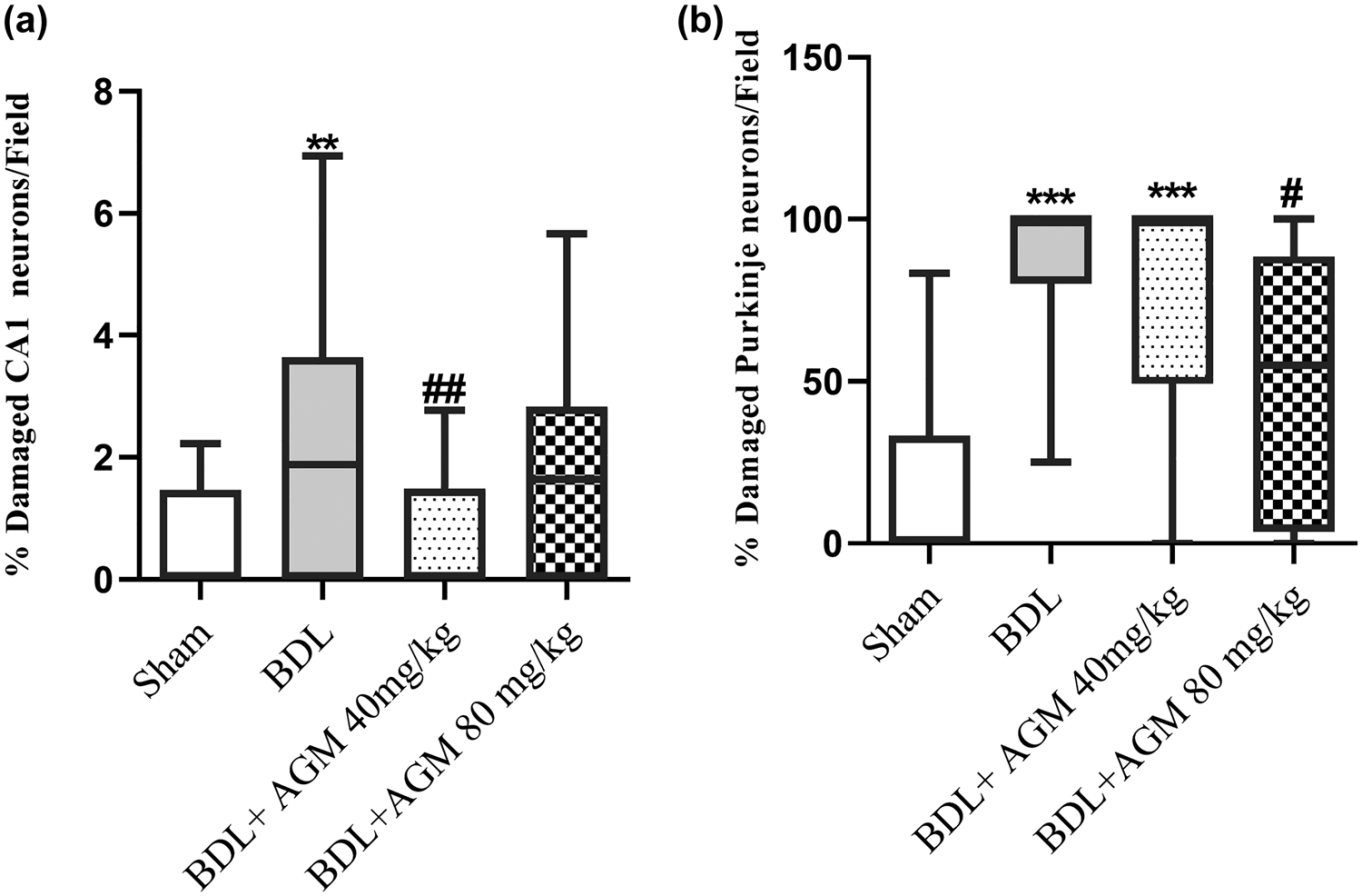
The percentages of damaged hippocampal CA1 (a) and cerebellar Purkinje neurons per field (b) were observed using the Nissl staining. ^**^ *p* <0.01, ^***^ *p* < 0.001 versus sham; ^#^ *p* < .05, ##*p* <0.01 versus bile duct ligation (BDL). Data are shown as box plots (Whiskers: min to max). Four animals/group, two slides/rat, and four fields/slide.

**FIGURE 9:**
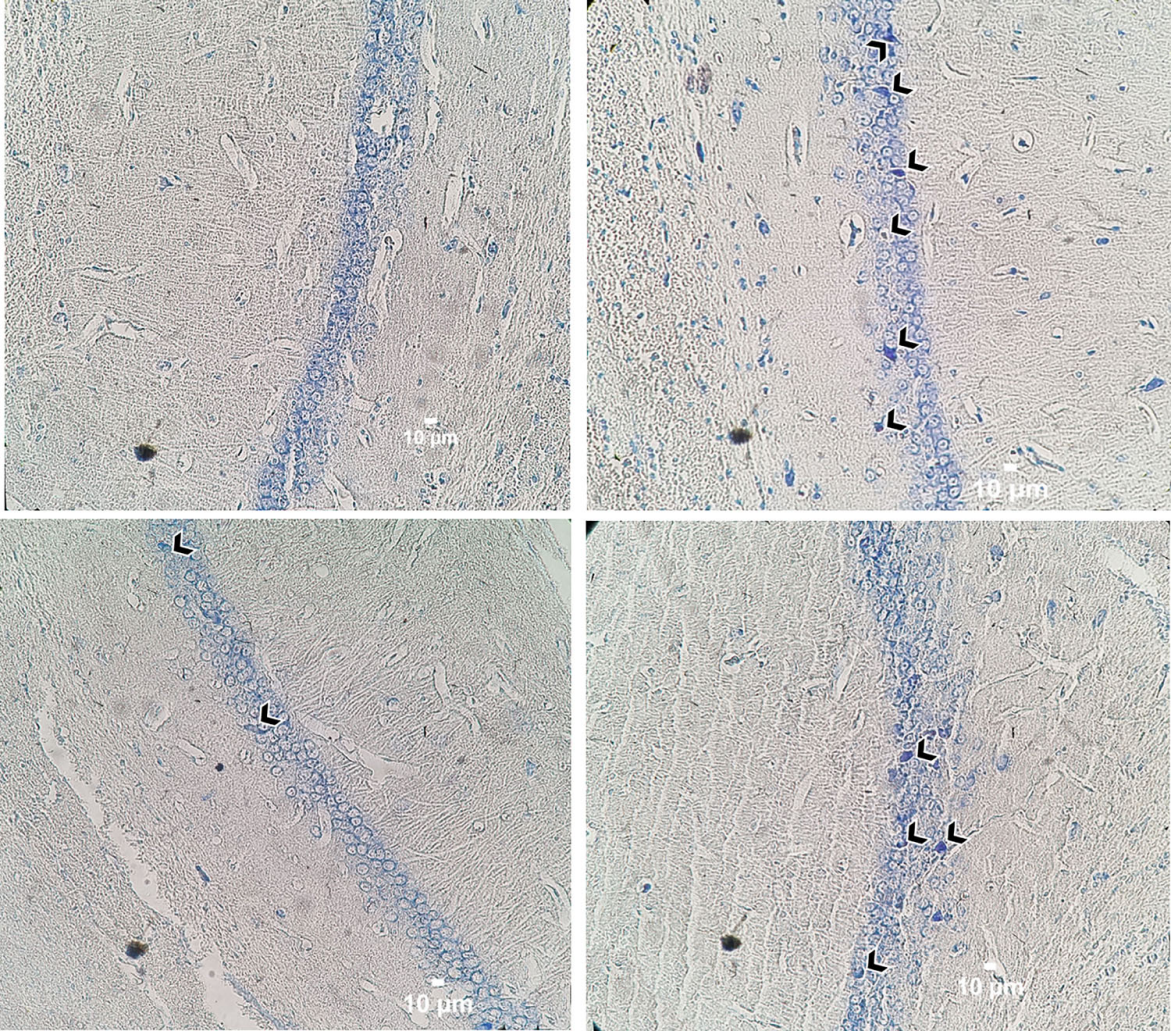
Nissl staining in the CA1 region of hippocampus in different groups. Head arrow shows damaged neurons (shrunken soma, pyknotic nucleus, and condensed cytoplasm). Bile duct ligation (BDL) induced insult to the neurons. Scale bar 10 μm.

**FIGURE 10:**
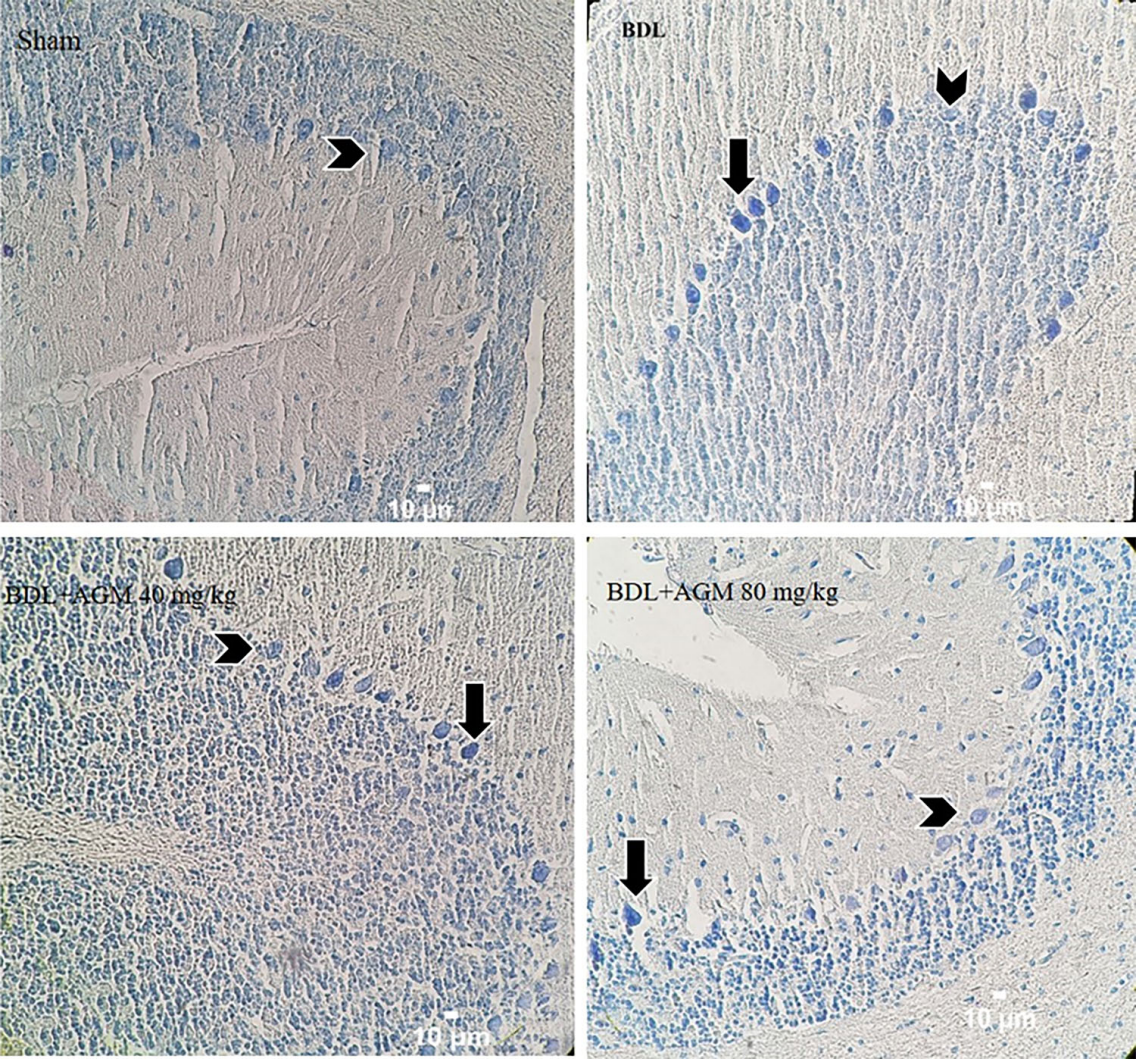
Nissl staining in the cerebellum in different groups. Arrows show damaged neurons and head arrows the healthy neurons. Bile duct ligation (BDL) induced insult to the neurons. Scale bar 10 μm.

To determine the effects of BDL and AGM on the neuronal damage in the CA1 region of hippocampus and cerebellum, Nissl staining was used. In BDL animals, there were more damaged neurons in both areas compared to the sham‐operated rats (CA1: Kruskal–Wallis statistic = 17.85, *p* = .005, Figure 8a; cerebellum; Kruskal–Wallis statistic = 34.01, *p* < .001). Treatment with 40 mg/kg AGM decreased the number of damaged neurons in the hippocampal CA1 region (*p* = .005, BDL+ AGM 40 mg/kg vs. BDL), whereas 80 mg/kg AGM decreased this value in the cerebellum (*p* = .01, BDL+ AGM 80 mg/kg vs. BDL). Figures 9 and 10 show the photomicrographs of the hippocampal CA1 region and Purkinje neurons in different groups, respectively. It is important to mention that the percentage of damaged neurons was obtained by counting damaged neurons for the four animals/group, two slides/rat, and four fields/slide. In some fields, the percentage of damaged neurons was 100%.

#### 3.5.2 Immunohistochemical staining of caspase‐3

**FIGURE 11:**
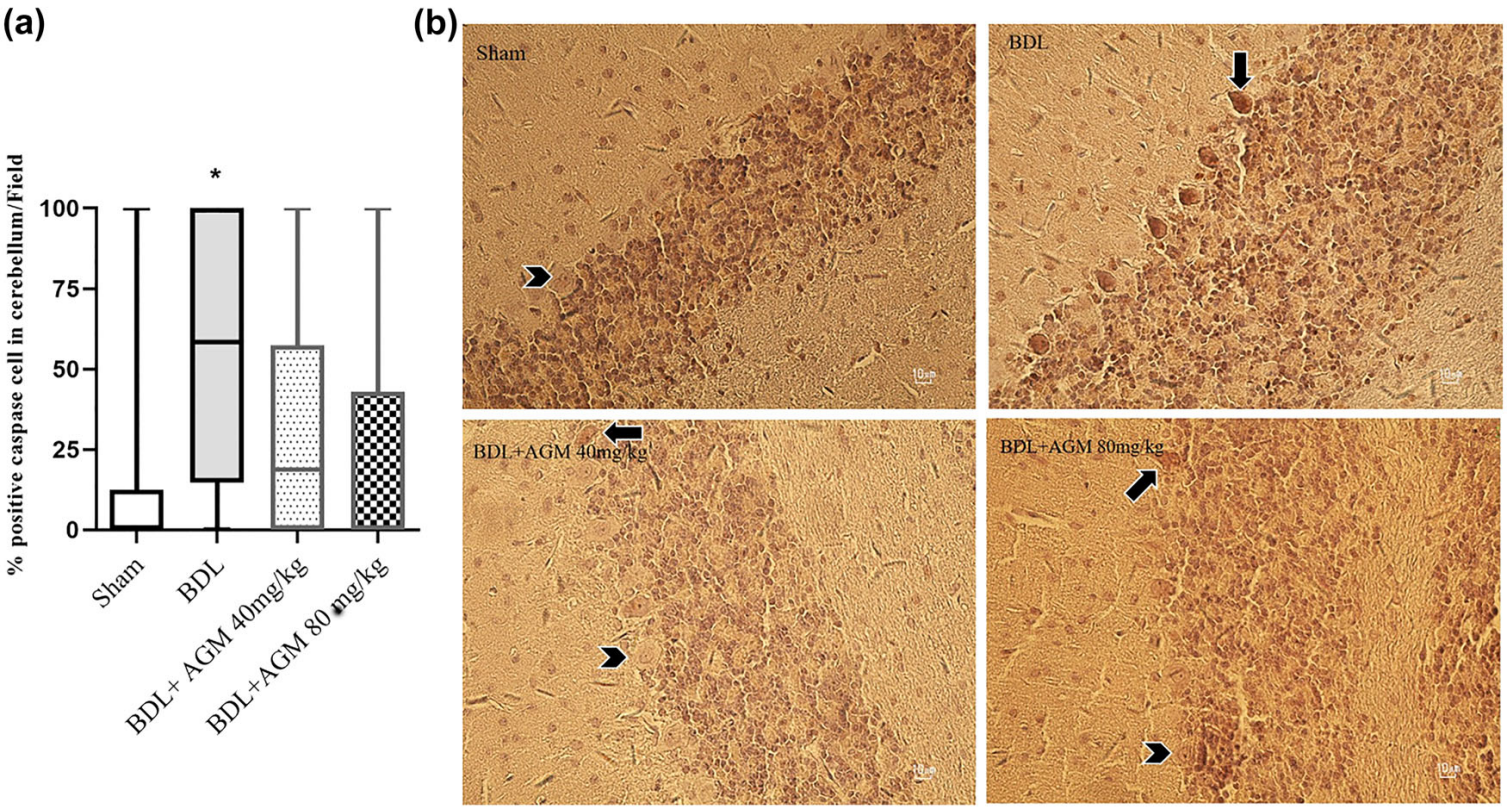
The expression of caspase‐3 per field in cerebellum in different groups. (a) The percentage of caspase‐3 positive cells (b) photomicrographs of caspase‐3 positive cells; ^*^ *p* < .05 versus sham; data are shown as box plots (Whiskers: min to max). Four animals/group, two slides/rat, and four fields/slide.

Positive caspase‐3 immunoreactivity is indicated by the presence of brown‐colored cells. Caspase‐3 expression in the cerebellum was significantly increased in the BDL group (Kruskal–Wallis statistic = 10.43, *p* = .014 vs. sham group, Figure 11). The differences in caspase‐3 expression were insignificant between BDL+ AGM 40 mg/kg versus BDL groups (*p* = .63), BDL+ AGM 40 mg/kg versus sham groups (*p* = .94), BDL+ AGM 80 mg/kg versus BDL groups (*p* = .08), BDL+ AGM 80 mg/kg versus sham groups (*p* > .99).

**FIGURE 12:**
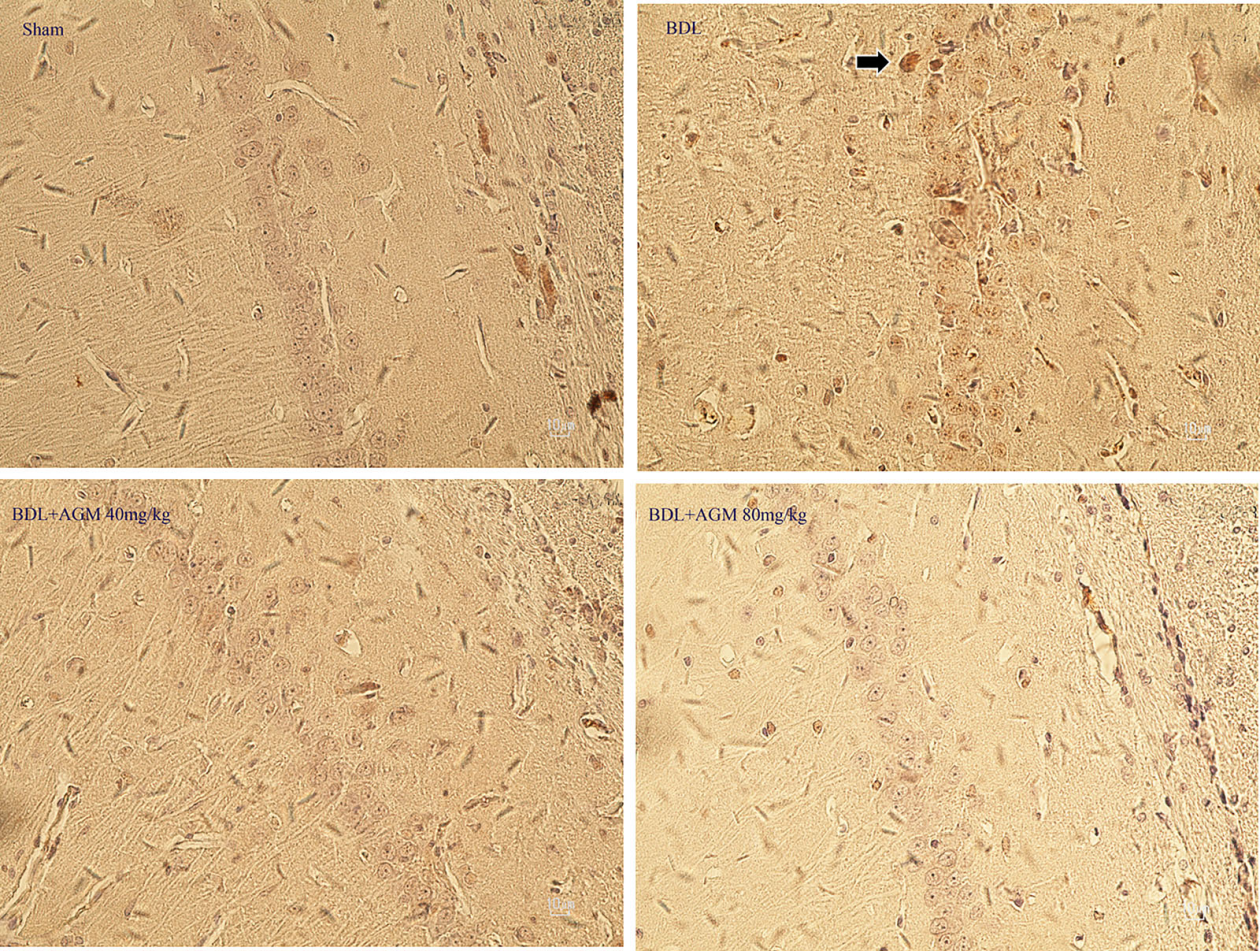
The expression of caspase‐3 in the CA1 region of the hippocampus, caspase‐3 positive cells are not seen in most subjects except only a few bile duct ligation (BDL) rats.

In the CA1 region of the hippocampus, caspase‐3 positive cells were not seen in most subjects except only a few BDL rats (Figure 12).

## 4 DISCUSSION

Several studies have revealed that AGM, as a metabolite of arginine, regulates urea synthesis and has antioxidative and anti‐inflammatory effects. The anxiolytic, antidepressant, and memory‐improving effects of AGM have been confirmed in several cognitive studies. Based on these findings and the etiology of HE, the present study aimed to examine the possible consequences of AGM on motor and cognitive performances in chronic cholestasis induced by BDL. Cell survival in the CA1 region of the hippocampus and cerebellum and liver function were evaluated to measure treatment efficacy.

In the present study, all BDL‐operated rats developed clinical symptoms of jaundice, such as dark urine, yellow skin, and mucous membrane. Chronic cholestasis damaged the hepatocytes and elevated the serum ALP, ALT, AST, total and direct bilirubin levels together with the AST/ALT ratio. AGM treatment in both doses decreased most of these elevated serum markers. Reduced motor activity, poor muscle strength, and balance were observed in the BDL rats. AGM rescued the balance and motor coordination on the rod without effects on motor learning and muscular strength. The results showed that the efficiency of both doses (40 and 80 mg/kg) of AGM was relatively identical in the biochemical and behavioral tests. In the NOR test, there were no differences in the performance of animals in different groups. The data from Nissl‐stained sections indicated that the number of damaged neurons in the CA1 region of the hippocampus and cerebellum was markedly high in the BDL rat model of HE and AGM protected the neurons. To further investigation, the expression of caspase‐3 protein, a key mediator of apoptosis, was examined through immunohistochemistry. Chronic cholestasis induced an increase in the activation of caspase‐3 in the Purkinje cells that was relatively reversed by AGM treatment, suggesting the antiapoptotic capability of AGM.

The protective effects of AGM treatment (5, 10, and 20 mg/kg, for 7 consecutive days after ligation) on acute hepatic injury were reported by a study, but the authors of that study did not evaluate the efficacy of AGM under chronic cholestasis which can present a wide spectrum of neuropsychiatric and neurological manifestations, including motor and cognitive impairment, named HE (Ommati et al., 2020). El‐Agamy et al. (2014) demonstrated that AGM can have hepatoprotective effects in a mouse model of hepatic inflammatory damage induced by d‐galactosamine and lipopolysaccharide.

Previous studies by El‐Sherbeeny et al. (2016) indicated that AGM can offer protection against nicotine‐induced hepatic damage. There is a study that introduces AGM as an effective drug for the treatment and prevention of liver ischemia reperfusion injury (Han et al., 2020). In the mentioned studies, reduced oxidative stress, nitric oxide, and inflammatory mediators were the proposed mechanism of action of AGM (Kim et al., 2016; Nissim et al., 2002).

It is important to note that arginine therapy in HE was recommended because of it stimulates ureagenesis, but it seems that arginine stimulates ureagenesis through metabolites. AGM, the decarboxylation product of arginine, irritates the hepatic content of NAG and ureagenesis and with respect to safety and efficacy in the treatment of cognitive and motor dysfunction can be a target for treating the HE (Nissim et al., 2002). Today, it is known as a popular dietary supplement to increase muscle mass in athletes and also for therapeutic purposes in combination medicine (Molderings & Haenisch, 2012).

In the current study, we did not observe any difference between BDL and sham‐operated rats in the NOR test. In consist with the results of our study, the results of the study by Cho et al. (2020) and Leke et al. (2013) showed that chronic cholestasis‐induced by BDL cannot change the performance of rodents on NOR test. NOR test is relatively less stressful, and animals do not receive an aversive stimulus. In the other words, it requires no external motivation, reward, or punishment and rodents explore the novel object as their innate desire for novelty. Furthermore, locomotor deficits induced by BDL may also be an important confounding factor in the NOR test. Our suggestion is that the test should not be used in future studies of HE or chronic BDL ().

Neuroprotection is another beneficial feature of AGM that makes it a therapeutic target in neurodegenerative diseases. The neuroprotective effects have been reported in numerous studies in both in vitro and vivo experimental models against the excitotoxicity induced by glutamate, glucocorticoids, amyloid β, and also in the animal model of spinal cord injury and traumatic brain injury (Neis et al., 2017; Zhu et al., 2008). Caspase‐3 results revealed that CA1 neurons are more resistant to BDL‐induced apoptosis than Purkinje cells; only a few apoptotic neurons were observed in this area. In the Nissl stained sections, the morphology of neurons in the hippocampal CA1 region and cerebellum was significantly different in BDL rats. AGM restored the CA1 neurons to normal at 40 mg/kg while recovered Purkinje cells at 80 mg/kg. The cerebellum histological results confirmed the motor coordination outcomes on the rotarod test.

There were some important methodological limitations in our study to estimate AGM levels in the brain and further study will be needed to determine whether the neuroprotective effects of AGM are related to increased AGM levels in the brain or other direct and indirect causes.

It is worth noting that, in our study, the AGM dosages were selected based on the existing literature of animal studies examining the influence of AGM following cognitive or liver deficits. In most of these studies, AGM (at dosages 20–100 mg/kg) was administered intraperitoneally or orally to adult rats. Moreover, previous preclinical studies showed that daily oral delivery of AGM is safe and increases brain and plasma AGM levels (Aglawe et al., 2021; Barua et al., 2019; Bergin et al., 2019; Miski et al., 2021).

Taken together, the present study suggests AGM as a potential therapeutic adjunct for HE. The beneficial effects of AGM used in this study on HE rats were most likely due to its beneficial effects on enhancing liver functions. The authors recommend future research examining the effects of AGM on NAG and comparing the effect of AGM versus arginine in HE animal models.

## CONFLICT OF INTEREST STATEMENT

The authors report that there are no conflict of interest to declare.

### PEER REVIEW

The peer review history for this article is available at https://publons.com/publon/10.1002/brb3.3124.

## Data Availability

The data that support the findings of this study are available from the corresponding author upon reasonable request.
